# Fabrication of palladium/graphene oxide composite by plasma reduction at room temperature

**DOI:** 10.1186/1556-276X-7-234

**Published:** 2012-04-26

**Authors:** Yue Yu, Yingzhi Li, Yunxiang Pan, Chang-jun Liu

**Affiliations:** 1 Advanced Nano Technology Center, School of Chemical Engineering and Technology, Tianjin University, 300072 Tianjin, China

**Keywords:** Nanoparticles, Composite materials, Palladium, Graphene oxide, Glow discharge, Plasma

## Abstract

Pd nanoparticles were fabricated on graphene oxide (GO) using a deposition-precipitation method with a glow discharge plasma reduction at room temperature. Argon was employed as the plasma-generating gas. The novel plasma method selectively reduces the metal ions. The graphene oxide has no change with this plasma reduction according to the Fourier transform infrared analysis. The Pd nanoparticles on the GO were uniformly distributed with an average diameter of 1.6 nm. The functional groups on the GO not only prevent Pd nanoparticles from further aggregation but also provide a strong hydrophilic property to the Pd/GO composite, which can form stable colloidal dispersions in water.

## Background

Graphene oxide (GO) is a highly oxidized layered graphene-based material with a large surface area and various functional groups such as -OH, -COOH, etc. [1-4]. These functional groups are chemically active so that GO can be decorated by various substances including biomolecules, metals and metal oxides [3,5,6]. GO can also be well dispersed in aqueous solutions due to the hydrophilic functional groups. The high solubility in water makes GO an ideal substrate for catalysts in water phase reactions. However, it is a challenge to reduce metal precursors on GO because GO is easily reduced with conventional reduction using NaBH_4_, N_2_H_4 _and ethylene glycol or irradiation method (microwave and laser pulse).

Here, we present a new fabrication method of Pd/GO composites using the room temperature glow discharge plasma reduction, which has been demonstrated to be a good strategy to reduce noble metal ions into nanoparticles [7-11]. The glow discharge plasma was shown not to affect the GO substrate. In order to maximize the loading efficiency and achieve high dispersion of Pd metal, deposition-precipitation was also carried out to anchor Pd precursor onto GO before the plasma reduction.

## Methods

GO was prepared by a modified Hummers method. Pd/GO composites were prepared by deposition of palladium hydroxide from hydrolysis of palladium chloride at pH 4 to approximately 10. In a typical synthesis, 4 mL aqueous solution of H_2_PdCl_4 _(1 × 10^-3 ^mol/L) was mixed with 4 mL aqueous solution of GO (1 mg/mL). NaOH (1 mol/L aqueous solution) was used to adjust the pH value of the H_2_PdCl_4 _and GO mixture. Then, the mixture was aged for 24 h before glow discharge plasma reduction. The details of glow discharge plasmas have been described previously [7,8].

The morphology of the sample was observed by transmission electron microscopy (TEM) and atomic force microscopy (AFM). TEM images were recorded with a Philips TECNAI G2F20 (Philips, Amsterdam, The Netherlands) system. AFM tests were performed by a Veeco Multimode Microscope V (Veeco Instruments Inc., Plainview, NY, USA) with spin coating of the sample on freshly cleaved mica substrates. The Fourier transform infrared (FT-IR) spectra were recorded using a Bruker Tensor 27 (Bruker Optics, Ettlingen, Germany) spectrometer with a resolution of 4 cm^-1^.

## Results and discussion

**Figure 1:**
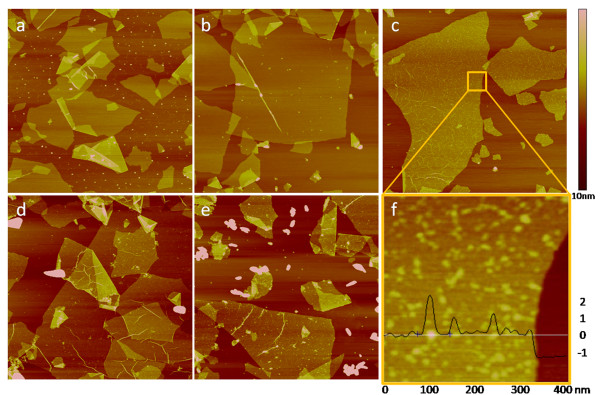
**Plasma-reduced Pd/GO samples prepared in different pH values**. (**a**) pH = 4, (**b**) pH = 5, (**c**) pH = 6, (**d**) pH = 7, (**e**) pH = 10 and (**f**) a zoom-in image of (c) with a cross-section analysis which shows the height of graphene and peaks arising from Pd nanoparticles. The scale of images (a) to (e) are 4 μm × 4 μm, and image (f) is 400 nm × 400 nm.

Figure 1 shows the AFM images of the plasma-reduced samples prepared in different pH values. The initial pH of the mixture H_2_PdCl_4 _and GO before adding any NaOH was 4 (Figure 1a). After 24 h of aging and the plasma reduction, particles were found mostly on the mica substrate and not on the GO, which means that Pd^2+ ^ions would not link with functional groups on GO at pH 4. By adding NaOH solution until pH reaches 5 (Figure 1b), a few particles were found on GO. Increasing the pH to 6 (Figure 1c,f) led to the formation of uniformly distributed fine particles anchored on GO substrates without free particles on mica substrates.

To see the particles more clearly, a zoom-in image (Figure 1f) and a cross-section analysis were made. The cross-section line went through one of the highest (brightest in color) particles in the whole image and a step between the GO and the mica substrate. It showed that the GO had a thickness of 1.3 nm. The highest particle was around 2 nm, and the majorities were around 1 nm in diameter. When the pH value was further increased to 7 and 10 (Figure 1d,e), large aggregations formed in the solution with little Pd loaded on GO. Thus, the best metal distribution with the highest metal loading efficiency occurred when pH was equal to 6. This sample (denoted as Pd/GO) was further studied by the following characterizations.

**Figure 2:**
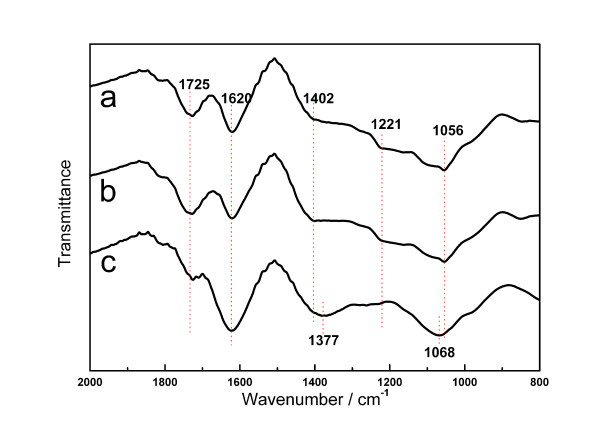
**FT-IR spectra**. (a) GO, (b) GO after glow discharge treatment and (c) Pd/GO.

FT-IR analysis was employed to determine the changes of functional groups on the GO surface in the glow discharge plasma reduction. Figure 2a presents the characteristic absorption bands of GO corresponding to the C = O carbonyl stretching (1,725 cm^-1^), O-H bend vibration (1,402 cm^-1^), C-OH stretching (1,221 cm^-1^) and C-O stretching of epoxide (1,056 cm^-1^) [5,12,13]. The spectrum also presents a peak at 1,620 cm^-1^. This was attributed to C = C ring vibrations throughout the carbon skeleton. However, the HOH bending vibrations also exist [12]. The spectrum of the GO after the glow discharge plasma reduction (Figure 2b) shows little difference compared with that of the untreated GO (Figure 2a), which proves that the glow discharge plasma has no effect on the functional groups of the GO. In the Pd/GO spectrum (Figure 2c), all characteristic absorption bands of the GO could be found in similar strength, indicating that the GO substrate was not reduced after the reduction of Pd nanoparticles. However, there were still tiny decreases in the absorption bands at 1,725, 1,221 and 1,056 cm^-1^. We can assume that the decreases were caused by the modification of the functional groups (C = O, C-OH and C-O) by the Pd nanoparticles.

**Figure 3:**
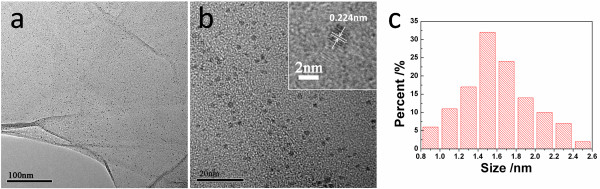
**TEM image**. (**a**) and (**b**) Pd/GO and (**c**) size distribution of Pd nanoparticles on GO.

TEM analysis was applied to determine the morphology of the Pd/GO sample. Figure 3 shows that Pd nanoparticles were uniformly dispersed on GO with an average particle diameter of 1.6 nm and a narrow distribution (standard deviation, SD = 0.37 nm). High-resolution TEM imaging (Figure 3b) of Pd NPs clearly showed the existence of lattice fringes with *d *= 0.224 nm, which could be attributed to the (111) planes of Pd. This result was consistent with the aforementioned AFM characterizations.

The pH effect of the Pd deposition process can be explained by the deposition-precipitation mechanism. GO sheets are highly negatively charged between pH 4 and 10, which can be attributed to the de-protonation of surface hydroxy and carboxy groups at the surface of GO [3-5]. Higher pH resulted in lower zeta potential and more de-protonation of surface groups. For palladium precursors in the solvent, with the addition of NaOH, palladium chloride would hydrolyze into polynuclear palladium (II) hydroxocomplexes (PHCs) with a diameter of 2 nm [14,15]. The PHC has a positively charged core by Na^+ ^cations and, thus, can be absorbed onto GO by electrostatic forces. In lower pH (4 and 5) (Figure 1a,b), PHCs were not formed, and electrostatic repulsion between GO substrate and PdCl_4_^2- ^complexes kept the palladium off the substrate.

## Conclusions

Pd nanoparticles with an average size of 1.6 nm are deposited on graphene oxide following a deposition-precipitation method and glow discharge plasma reduction at room temperature. The pH value has a significant effect on the fabrication of Pd/GO composites. The glow discharge plasma can efficiently reduce Pd precursors while maintaining GO unreduced. Pd/GO composite is successfully fabricated with high dispersion of metal nanoparticles.

## Abbreviations

GO: Graphene oxide; AFM: Atomic force microscopy; FT-IR: Fourier transform infrared; TEM: Transmission electron microscopy; PHC: Palladium (II) hydroxocomplex.

## Competing interests

The authors declare that they have no competing interests.

## Authors' contributions

YY synthesized and characterized the Pd/GO samples and wrote the manuscript. YZL prepared the raw GO material, YXP conceived and designed the experiments, and CJL coordinated the study. All authors read and approved the final manuscript.
